# Acylated and unacylated ghrelin do not directly stimulate glucose transport in isolated rodent skeletal muscle

**DOI:** 10.14814/phy2.13320

**Published:** 2017-07-04

**Authors:** Daniel T. Cervone, David J. Dyck

**Affiliations:** ^1^ Department of Human Health and Nutritional Sciences University of Guelph Ontario Canada

**Keywords:** Ghrelin, glucose transport, rat, skeletal muscle

## Abstract

Emerging evidence implicates ghrelin, a gut‐derived, orexigenic hormone, as a potential mediator of insulin‐responsive peripheral tissue metabolism. However, in vitro and in vivo studies assessing ghrelin's direct influence on metabolism have been controversial, particularly due to confounding factors such as the secondary rise in growth hormone (GH) after ghrelin injection. Skeletal muscle is important in the insulin‐stimulated clearance of glucose, and ghrelin's exponential rise prior to a meal could potentially facilitate this. This study was aimed at elucidating any direct stimulatory action that ghrelin may have on glucose transport and insulin signaling in isolated rat skeletal muscle, in the absence of confounding secondary factors. Oxidative soleus and glycolytic extensor digitorum longus skeletal muscles were isolated from male Sprague Dawley rats in the fed state and incubated with various concentrations of acylated and unacylated ghrelin in the presence or absence of insulin. Ghrelin did not stimulate glucose transport in either muscle type, with or without insulin. Moreover, GH had no acute, direct stimulatory effect on either basal or insulin‐stimulated muscle glucose transport. In agreement with the lack of observed effect on glucose transport, ghrelin and GH also had no stimulatory effect on Ser^473^ AKT or Thr^172^ AMPK phosphorylation, two key signaling proteins involved in glucose transport. Furthermore, to our knowledge, we are among the first to show that ghrelin can act independent of its receptor and cause an increase in calmodulin‐dependent protein kinase 2 (CaMKII) phosphorylation in glycolytic muscle, although this was not associated with an increase in glucose transport. We conclude that both acylated and unacylated ghrelin have no direct, acute influence on skeletal muscle glucose transport. Furthermore, the immediate rise in GH in response to ghrelin also does not appear to directly stimulate glucose transport in muscle.

## Introduction

Ghrelin is a gut‐derived hormone with central effects controlling appetite. Its production and release increases exponentially prior to meals and returns to basal levels immediately postprandially (Kojima et al. 1999; Liu et al. 2008). Ghrelin can be posttranslationally acylated by ghrelin‐*O*‐acyl‐transferase (GOAT) and unacylated by serum esterases (Gutierrez et al. 2008; Yang et al. 2008; Satou and Sugimoto 2012). It exists primarily unbound and unacylated, although many of ghrelin's effects can be attributed to its acylated form (Patterson et al. 2005; Liu et al. 2008). Acylated ghrelin (AG) acts through GHS‐R1a in nonmuscle peripheral tissues, and potentially the corticotropin‐releasing factor (CRF‐2) receptor in muscle (Filigheddu et al. 2007; Gershon and Vale 2014). Unacylated ghrelin (UnAG) is believed to act through an alternate receptor (Lear et al. 2010; Gershon and Vale 2014) in peripheral tissues such as muscle. Ghrelin's effects on peripheral glucose utilization have not been extensively studied and are unclear. Several studies (St‐Pierre et al. 2007; Vestergaard et al. 2008a, 2008b, 2011) suggest that AG infusion acutely impairs the clearance of glucose from the blood. Furthermore, ghrelin KO and ghrelin receptor KO mice are protected from the negative effects of a high fat diet on glucose tolerance and insulin sensitivity (Qi et al. 2011). In contradiction to these findings, AG injection has recently been shown to improve blood glucose clearance during an oral glucose tolerance test in mice, which was linked to the ability of AG to stimulate the secretion of glucagon‐like peptide‐1 (GLP‐1) (Gagnon et al. 2014) an intestinally released incretin which serves to potentiate insulin release (Brubaker 2010; Campbell and Drucker 2013; Gagnon et al. 2014). Taken together, there is controversy as to the in vivo effects of ghrelin on peripheral glucose metabolism.

A potentially confounding factor when examining the effects of in vivo ghrelin administration is the change in circulating concentrations of secondary hormones. For example, AG is well known to stimulate growth hormone (GH) release (Kojima et al. 1999; Tschöp et al. 2000; Wren et al. 2000). Growth hormone has been shown to stimulate adipose lipolysis (Fain et al. 2008), which may in turn alter (i.e., reduce) peripheral glucose utilization. However, AG has also been demonstrated to acutely increase GLP‐1 and subsequently insulin (Gagnon et al. 2014), which conversely could lead to increased peripheral glucose transport. The direct, isolated effects of ghrelin isoforms on peripheral insulin‐sensitive tissues such as skeletal muscle, in the absence of these secondary hormonal changes, have not been extensively examined. Such an examination is required to fully understand the metabolic role of ghrelin in tissues such as skeletal muscle.

The effects of ghrelin on skeletal muscle metabolism have not been extensively investigated and are inconsistent. One study reported increases in key insulin‐responsive proteins (AKT, glycogen synthase kinase, GLUT4) in oxidative muscle (Barazzoni et al. 2007) in response to in vivo AG injections over a period of several days in rats, while others exhibit no alteration in AKT following injection or infusion, in both rats and humans (Vestergaard et al. 2008a; Barazzoni R et al. 2011). There is also some, albeit limited evidence for a direct effect of ghrelin on isolated muscle cells. In C2C12 myocytes, direct exposure to AG for 24 h increases GLUT4 translocation to the cell surface and glucose transport (Gershon and Vale 2014). However, to date there has been no examination of ghrelin's direct and isolated effect on glucose transport in mature, intact skeletal muscle. Given ghrelin's rise just prior to the consumption of food, it is tempting to consider whether ghrelin could prime tissues such as skeletal muscle in the preparation for the delivery of glucose, making them more responsive to insulin. To our knowledge, this potential role for ghrelin has largely been ignored. Therefore, the overall aim of this study was to determine the direct effects of ghrelin's two main isoforms on the transport of glucose in isolated rat skeletal muscle, in the presence and absence of insulin. More specifically, we hypothesized that ghrelin would directly stimulate glucose transport in skeletal muscle in the presence, but not the absence of insulin.

## Methods

### Animals

All procedures were approved by the Animal Care Committee at the University of Guelph and followed Canadian Council of Animal Care guidelines. Male Sprague Dawley rats were purchased from Charles River Laboratories (Québec, ON, Canada) at approximately 6 weeks of age (~200 g). Rats were given free access to water and a regular chow diet, ad libitum. Food was provided until approximately 3 h prior to experiments to ensure that animals were in the fed state for terminal surgeries. Rodents were anesthetized with an intraperitoneal injection of pentobarbital sodium (6 mg/100 g body mass) prior to all surgical procedures.

### Materials and reagents

Reagents, molecular weight markers, and nitrocellulose membrane were purchased from BioRad (Mississauga, ON, Canada). Western Lightning Plus enhanced chemiluminescence (ECL) was purchased from Perkin‐Elmer (NEL105001EA). The following primary antibodies were purchased from Cell Signaling: phospho‐AKT Ser^473^ (Cat. # 4060), phospho‐AKT Thr^308^ (Cat. # 9275S), phospho‐CaMKII Thr^287^ (Cat. # 12716), phospho‐STAT5b Tyr^694^ (Cat. # 4322), total AKT (Cat. # 9272S), total AMPK (Cat. # 2603S), total CaMKII (Cat. # 4436S) and total STAT5 (Cat. # 9363T). NP40 cell lysis buffer was acquired from Life Technologies and PMSF and protease inhibitor cocktail were obtained from Sigma (Cat. # 78830 and 9599). Insulin (Humulin rDNA origin) was purchased from Eli Lilly (Toronto, ON, Canada).

Incubation buffer (Krebs–Henseleit base) constituents were purchased from Sigma‐Aldrich and include: d‐glucose (Cat. #G‐8270), d‐mannitol (Cat. #M‐9546), sodium pyruvate (Cat. #P8574) and 3‐methyl‐*O*‐glucopyranose (Cat. #M‐4879). Growth hormone was obtained from Abcam (Cat. #ab68388). Acylated (Cat. #H‐4862) and unacylated (Cat. #H‐6264) ghrelin were sourced from Bachem (Torrance, CA). Radioactive ^14^C‐mannitol (Cat. #CFA‐238 – 250 *μ*Ci/1250 *μ*L) and 3‐*O*‐[^3^H]methyl‐d‐glucose (Cat. #ART‐126 – 1 mCi/1000 *μ*L) tracers were purchased from American Radiolabeled Chemicals (St. Louis, MO).

### Ghrelin stability verification

To our knowledge, the stability of either ghrelin isoform in an incubation medium has not been reported. To test this, incubation buffer samples were taken at *t* = 0, 30, 60 and 120 min and analyzed for ghrelin concentration using a commercially available kit (EMD Millipore Cat. EZRGRT‐91K) according to manufacturer instructions. Accuracy and intra‐assay comparisons were validated, using two quality control standards (run in triplicate) provided with each kit. Buffer samples were assayed in duplicate.

### Glucose transport assays

Glucose transport assays in isolated skeletal muscle were carried out as previously described (Bruce et al. 2005; Mullen et al. 2007; Thrush et al. 2008; Ritchie et al. 2011). Briefly, media were pregassed with 95% O_2_ and 5% CO_2_ and heated in a shaking water bath at 30°C. One muscle strip for each condition within a set of experiments was obtained from a single animal, as each muscle can be stripped into 2–3 viable, 20–30 mg sections. Muscles were allowed to float in medium and did not have tension applied. Soleus (oxidative) and extensor digitorum longus (glycolytic) muscles were stripped lengthwise, excised with tendons intact and preincubated in medium (M1)‐containing 8 mmol/L d‐glucose and 32 mmol/L d‐mannitol for 1 h. Next, muscles were carefully transferred to vials containing a medium (M2) of 36 mmol/L d‐mannitol and 4 mmol/L pyruvate to undergo two separate 15 min washes. Lastly, muscles were transferred to vials which contained a medium (M3) of 8 mmol/L 3‐methyl‐*O*‐glucopyranose, 28 mmol/L d‐mannitol, pyruvate 4 mmol/L, 0.5 *μ*L/mL 3‐*O*‐[^3^H]methyl‐d‐glucose, 1.0 *μ*L/mL ^14^C‐mannitol for 1 h. Acylated or UnAG was added to all media (M1, 2 and 3) for non‐insulin stimulated glucose transport determinations. To determine whether AG or UnAG had a priming effect on insulin‐stimulated glucose transport, AG or UnAG were added to preincubation and wash incubations only (M1 and 2), but not the final transport medium (M3) in an attempt to mimic the physiological state of high insulin and low/declining ghrelin following meal consumption. To determine growth hormone's (GH) effects on glucose transport, GH was added to the wash and final transport incubation (M2 and M3).

For insulin‐stimulated glucose transport assays, insulin was added to both wash media (M2) as well as the final transport incubation (M3) at either 0.5 mU/mL (moderate) (Tishinsky et al. 2014) or 10 mU/mL (high) (Ritchie et al. 2011) concentrations. Following incubation, muscles were trimmed of their tendons, blotted and weighed. Muscles were then boiled and solubilized for ~10 min in 1 mL of 1 mol/L NaOH and vortexed periodically throughout. A 200 *μ*L sample of muscle digest was added to scintillation vials with 5 mL of Cytoscint scintillation fluorescent cocktail (MP Biomedicals ‐ via Cedarlane ‐ Burlington, ON, Canada) and swirled to ensure proper mixing. Samples were allowed to quench overnight in darkness and then counted for 5 min per sample, using a Beckman‐Coulter LS5600 liquid scintillation counter, and were considered acceptable when the detected Lumex percentage (random counts per minute) was less than 5%. Glucose transport was calculated as the accumulation of intracellular labeled glucose as we have previously reported (Ritchie et al. 2011).

### Ghrelin, growth hormone and insulin signaling

Incubations for determining hormonal signaling were carried out so as to closely mimic our glucose transport protocol. In short, muscle strips from soleus and EDL were removed from one leg and incubated for 1 h in preincubation buffer, then transferred to a second (untreated) buffer for 15 min, as described above. Strips from the contralateral leg were also excised, preincubated for 1 h, and then transferred to a second experimental buffer containing various treatments (i.e. 150 ng/mL AG or UnAG, 250 ng/mL GH, 10 mU/mL insulin) for 15 min. This brief window was chosen to detect any rapid transient changes in signaling proteins (e.g., phosphorylation of AKT, AMPK, etc.). All tissues were immediately frozen in liquid nitrogen and then stored at −80°C until further processing for western blotting.

### Western blotting

Muscles were chipped if required under liquid nitrogen into 20–30 mg pieces, and then placed directly into homogenization tubes containing lysis beads and stored in liquid nitrogen. Cell lysis buffer supplemented with protease inhibitor and PMSF were then added, and samples were homogenized and centrifuged at 4°C. Supernatant was then removed and transferred to a new tube. BCA assays were performed to determine protein content from homogenized samples for subsequent sample preparation and western blots (Smith et al. 1985).

For western blots, equal amounts of sample protein (p‐AKT/AMPK: 20 *μ*g; p‐CaMKII: 25 *μ*g; p‐STAT5: 30 *μ*g) were loaded onto 10% gels, as we have published previously (Mullen et al. 2007; Ritchie et al. 2011). Samples were then transferred for 1 h at 0.2 A onto nitrocellulose membranes and blocked in nonfat skim milk powder and TBST. Membranes were then incubated with primary antibody (1:1000 p‐AKT, p‐AMPK, p‐CaMKII and all total proteins; 1:500 p‐STAT5) diluted in 5% BSA and incubated overnight at 4°C. Afterwards, membranes were washed with TBST and exposed to secondary (anti‐rabbit) antibody (1:2000) for 1 h at room temperature and then washed again (2x TBST, 1x TBS). Bands were visualized, using ECL and quantified using densitometry on Alpha Innovate Software. Ponceau staining was used as a loading control.

### Statistics

All data are expressed as mean ± standard error. A repeated measure one‐way analysis of variance (ANOVA) was performed for all experiments examining the effects of ghrelin and growth hormone, and their interaction with insulin, on glucose transport and signaling proteins. If significance was detected with the ANOVA, multiple comparisons were assessed using Tukey's post hoc test. A paired student's *t*‐test was performed for experiments examining growth hormone effects (independent of insulin) on glucose transport and signaling proteins. In all transport figures, letters are used to denote statistical significance, such that groups sharing a letter are not significantly different. Data were considered significant at *P* < 0.05.

## Results

### AG and UnAG remain stable in incubation buffer for 2 h

Despite the prevalence of studies which use cell (e.g., adipocyte, myocyte) incubations to assess ghrelin's effects, there has not been any previously reported validation of ghrelin's stability in such a preparation. Thus, prior to commencing glucose transport incubations, it was first confirmed that AG and UnAG, added to produce a final buffer concentration of 4 and 12 ng/mL, respectively, remained stable at 30°C for a 2 h period. Sampling was done at *t* = 0, 30, 60 and 120 min and there were no indications to suggest that ghrelin was declining in the incubation solution. Acylated and UnAG ghrelin concentrations at 0, 30, 60 and 120 min were as follows: AG; 3.6, 3.4, 3.1 and 3.4 ng/mL and UnAG; 9.3, 11.2, 12.5 and 13.1 ng/mL.

### AG and UnAG do not stimulate muscle glucose transport ex vivo, either in the presence or absence of insulin

**Figure 1:**
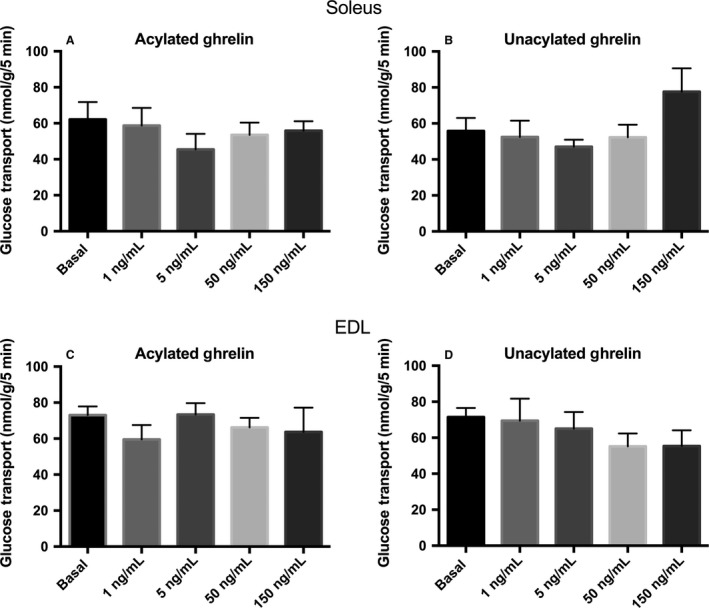
Effect of acylated and unacylated ghrelin on glucose transport in the absence of insulin. 3‐*O*‐[^3^H]methyl‐d‐glucose transport (nmol/g/5 min) in incubated oxidative soleus (A and B) and glycolytic EDL (C and D) muscles with ghrelin alone. Data were analyzed using a repeated measures one‐way ANOVA (*n* = 10). There were no statisically significant differences among groups.

Over a wide range of concentrations (1–150 ng/mL), neither AG nor UnAG had any significant stimulatory effect on the rate of 3‐*O*‐[^3^H]methyl‐d‐glucose transport independent of insulin, within oxidative soleus (Fig. 1A and B) or glycolytic EDL skeletal muscle (Fig. 1C and D).

**Figure 2:**
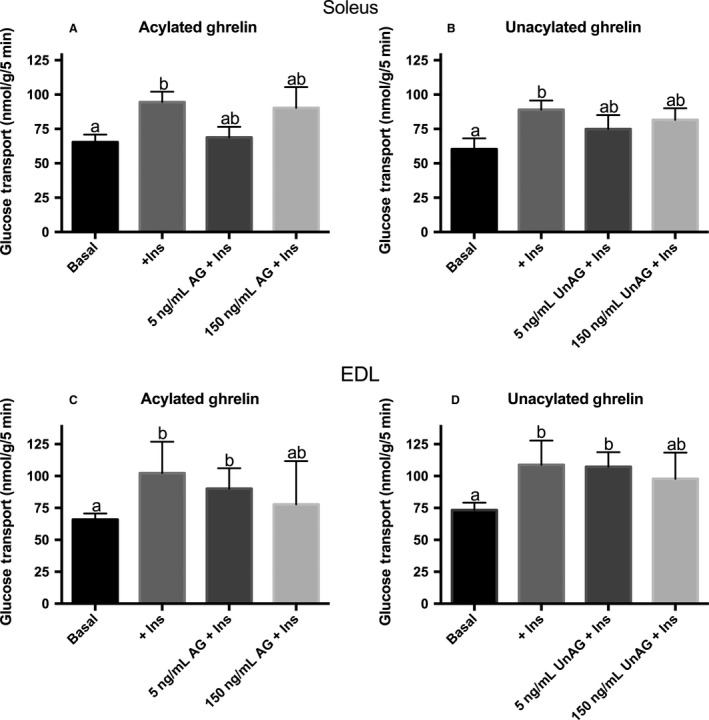
Effect of acylated and unacylated ghrelin on glucose transport with a moderate dose of insulin. 3‐*O*‐[^3^H]methyl‐d‐glucose transport in oxidative soleus (A and B) and glycolytic EDL (C and D) muscles following preincubation with either moderate insulin (0.5 mU/mL) alone or moderate insulin in conjunction with ghrelin. Groups sharing a letter are not statistically different. AG, acylated ghrelin; UnAG, unacylated ghrelin; Ins, insulin; EDL, extensor digitorum longus. Data were analyzed using a repeated measures one‐way ANOVA (*n* = 9–10).

In both soleus and EDL, a moderate dose of insulin (0.5 mU/mL) significantly increased glucose transport by 44% and 52% (*P* < 0.05), respectively, compared to control (Fig. 2AD). However, glucose transport in the presence of insulin was not increased further by physiological and supraphysiological concentrations of AG (Fig. 2A and C), or UnAG (Fig. 2B and D) in either oxidative or glycolytic muscle. It should also be noted that in soleus, neither of the insulin conditions with ghrelin (Fig. 2A and B) was statistically different from the basal condition. Thus, it might be argued that ghrelin prevented insulin from stimulating glucose transport, although this is complicated by the fact that none of the insulin conditions (insulin, insulin + AG, insulin + UnAG) were statistically different from each other. This is also true in EDL with the highest concentration of AG and UnAG (Fig. 2C and D). Taken together, while it is somewhat tenuous to state that ghrelin decreased insulin‐stimulated glucose transport, our data are very clear that the ghrelin did not increase glucose transport, either in the presence or absence of insulin, which was the specific hypothesis that we were testing.

**Figure 3:**
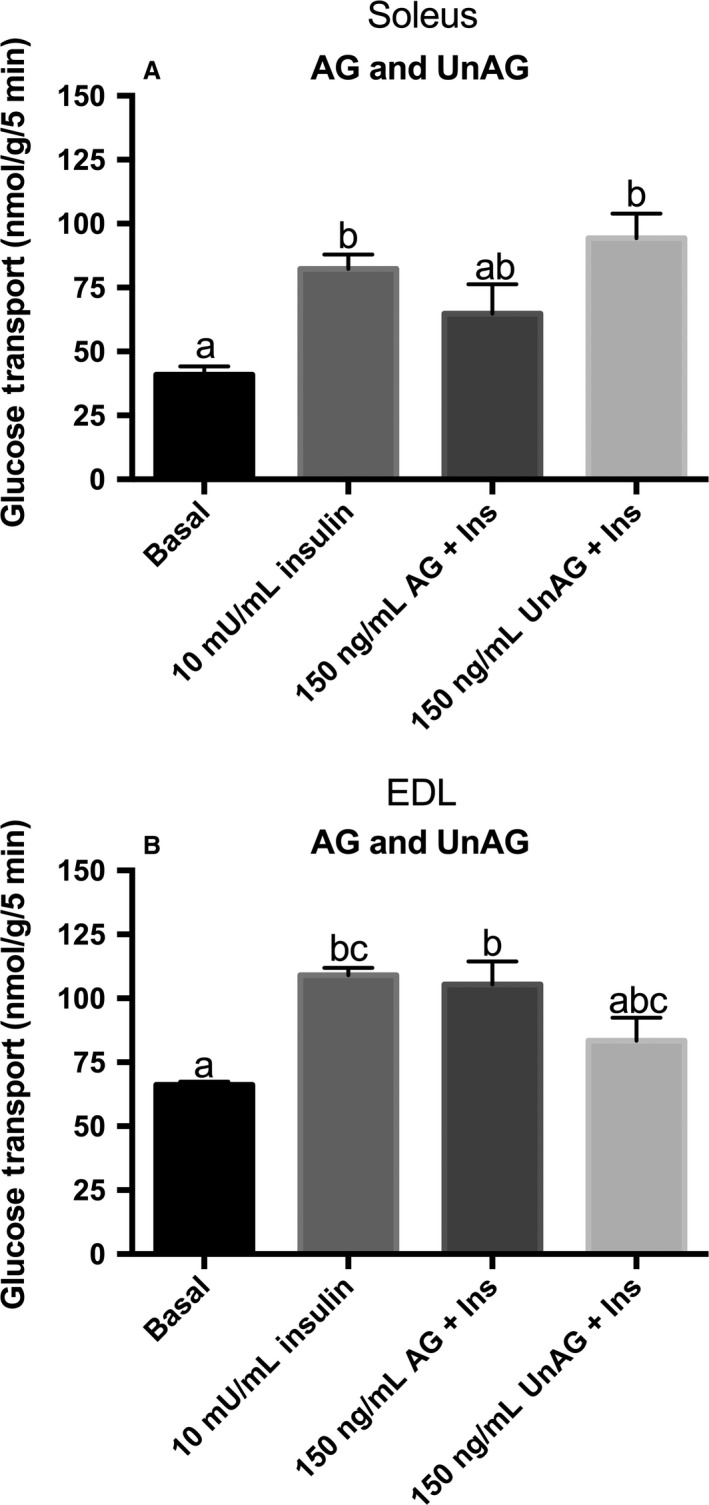
Effect of acylated and unacylated ghrelin with a high dose of insulin on glucose transport. 3‐*O*‐[^3^H]methyl‐d‐glucose transport in oxidative soleus (A) and glycolytic EDL (B) muscles following preincubation with either high insulin (10 mU/mL) alone or high insulin in conjunction with ghrelin. Groups sharing a letter are not statistically different. AG, acylated ghrelin; UnAG, unacylated ghrelin; Ins, insulin; EDL, extensor digitorum longus. Data were analyzed using a repeated measures one‐way ANOVA (*n* = 6).

High dose insulin‐stimulation resulted in a twofold increase (*P* < 0.05) in the rate of glucose transport in soleus (Fig. 3A) and a 65% increase (*P* < 0.05) in EDL (Fig. 3B). As previously observed, neither AG nor UnAG significantly increased glucose transport in the presence of insulin in either muscle. Again, it is perhaps worthwhile noting that glucose transport in the presence of insulin was not statistically different from the basal condition with a high dose of AG in soleus, and UnAG in EDL.

### AKT Ser^473^ and Thr^308^ phosphorylation is increased following insulin, but not AG or UnAG incubation

**Figure 4:**
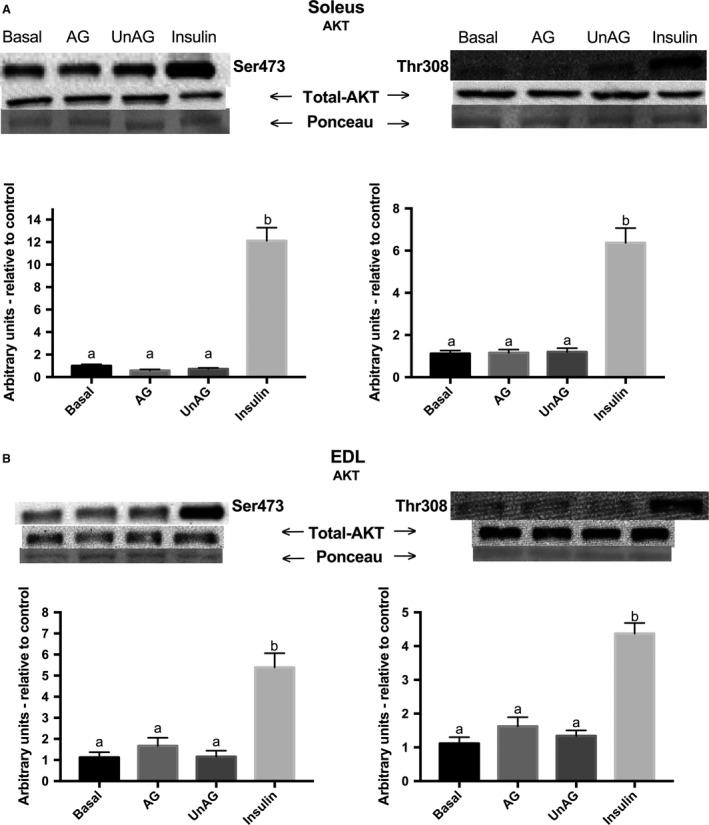
Effect of acylated and unacylated ghrelin, and insulin on the phosphorylation of AKT Ser^473^ and Thr^308^ protein content following incubation with ghrelin (150 ng/mL) or insulin (10 mU/mL) in (A) oxidative soleus and (B) glycolytic EDL muscles. Groups sharing a letter are not statistically different. AG, acylated ghrelin; UnAG, unacylated ghrelin; EDL, extensor digitorum longus. Data were analyzed using a repeated measures one‐way ANOVA (*n* = 10).

There was a robust increase in the phosphorylation of the insulin signaling protein AKT at two of its activating sites (Ser^473^ and Thr^308^) in both soleus (7.1 ± 0.5 fold; *P* < 0.05) and EDL (5.4 ± 0.24 fold; *P* < 0.05), when subjected to a high dose (10 mU/mL) of insulin (Fig. 4A and B). However, AG and UnAG had no acute effect on the activation of AKT, which is in agreement with the lack of a stimulatory effect of ghrelin on glucose transport.

### CaMKII Thr^287^ phosphorylation is significantly increased following AG incubation, but only in glycolytic skeletal muscle

**Figure 5:**
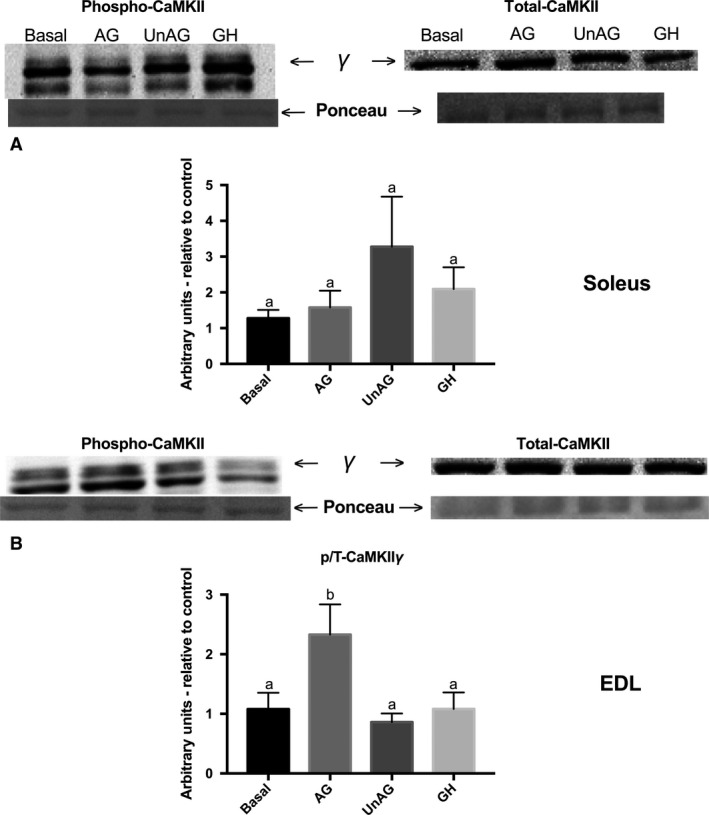
Effect of acylated and unacylated ghrelin, and growth hormone on p‐CaMKII *γ* (Thr^287^) protein content following incubation with ghrelin (150 ng/mL) or GH (250 ng/mL) in (A) oxidative soleus and (B) glycolytic EDL muscles. Groups sharing a letter are not statistically different. AG, acylated ghrelin; UnAG, unacylated ghrelin; GH, growth hormone; EDL, extensor digitorum longus. Data were analyzed using a repeated measures one‐way ANOVA (*n* = 10).

Acylated ghrelin signals via GHS‐R1a in several peripheral tissues, although its levels in skeletal muscle have been considered to be negligible (Gnanapavan et al. 2002). Rather, AG is thought to potentially interact with the corticotropin‐releasing factor (CRF‐2) receptor in muscle (Filigheddu et al. 2007; Gershon and Vale 2014), while UnAG does not have an identified receptor, although it is not a ligand for GHS‐R1a and has been shown to have metabolic effects in peripheral tissues distinct from AG (Filigheddu et al. 2007; Lear et al. 2010). Although UnAG signaling/receptor interactions remain largely unknown, classical ghrelin signaling via GHS‐R1a results in the transient release of calcium (Howard et al. 1996; Kojima et al. 1999). However, whether ghrelin activates similar signaling pathways in isolated mature skeletal muscle, has not been examined to our knowledge. To this end, we examined CaMKII phosphorylation, the major multifunctional calmodulin‐kinase in skeletal muscle (Rose et al. 2006), as an initial attempt to detect ghrelin signaling in skeletal muscle. CaMKII (*δ*: ~57 kDa, *γ*: ~62 kDa and *β*_M_: ~72 kDa) phosphorylation was detected as three bands, consistent with what has been previously reported in skeletal muscle (Rose et al. 2006). During pilot analysis, the lower two (*δ* and *γ*) bands followed a loading dose–response relationship and were therefore quantified for study purposes. Since we did not have antibody for total CaMKII*δ* (from Cell Signaling), only p‐CamKII*γ* is shown expressed relative to total *γ* protein. Interestingly, CaMKII phosphorylation was not increased in oxidative soleus muscle (Fig. 5A). However, the content of p‐CaMKII*δ* (not shown) and p‐CaMKII*γ* was increased in EDL, but only following incubation with AG (Fig. 5B), serving as a positive control for ghrelin signaling in this muscle.

### AMPK phosphorylation (Thr^172^) is unchanged following incubation with AG, UnAG and GH

**Figure 6:**
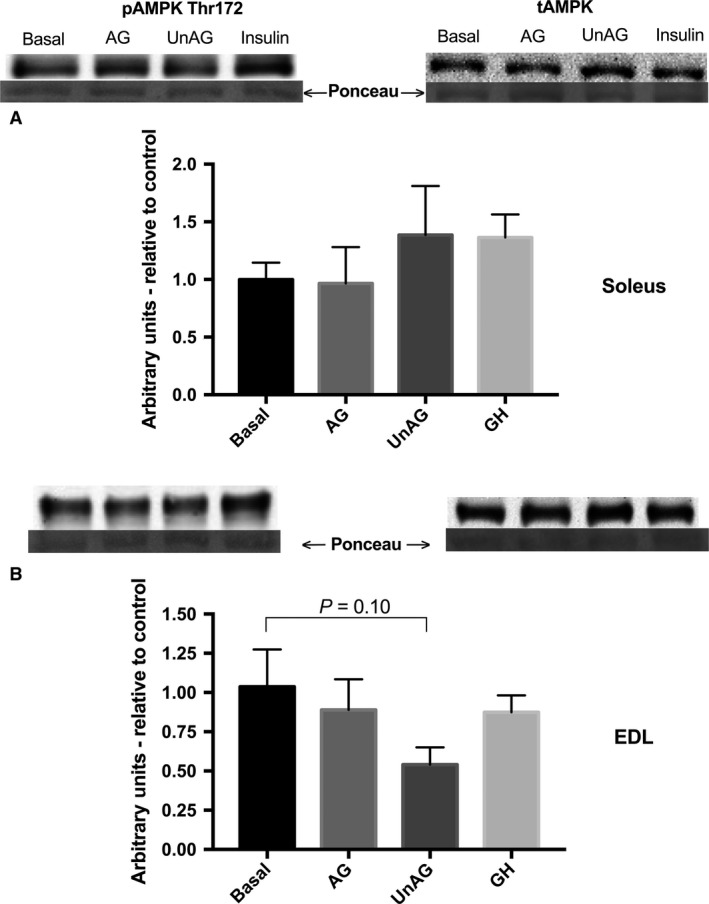
Effect of acylated and unacylated ghrelin, and growth hormone on the phosphorylation of AMPK Thr^172^ protein content following incubation with ghrelin (150 ng/mL) or GH (250 ng/mL) in (A) oxidative soleus and (B) glycolytic EDL muscles. There were no statisically significant differences among groups. AG, acylated ghrelin; UnAG, unacylated ghrelin; GH, growth hormone; EDL, extensor digitorum longus. Data were analyzed using a repeated measures one‐way ANOVA (*n* = 10).

Consistent with previous findings with ghrelin in rodent skeletal muscle (Kola et al. 2005), there was no observed changes in the state of AMPK activation in soleus or EDL following ghrelin or GH treatment (Fig. 6A and B).

### Incubation with GH has no effect on basal or insulin‐stimulated glucose transport, or activation of insulin signaling protein, AKT (Ser473 and Thr^308^)

**Figure 7:**
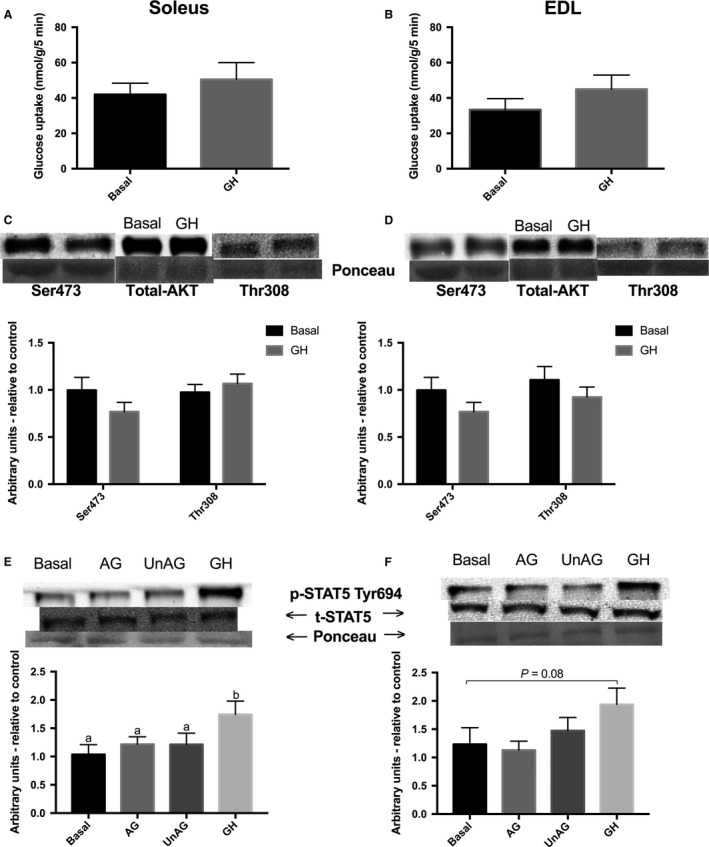
Effect of growth hormone on glucose uptake and phosphorylation of AKT Ser^473^ and Thr^308^, and STAT5 Tyr^694^. 3‐*O*‐[^3^H]methyl‐d‐glucose uptake (nmol/g/5min) in incubated oxidative soleus (A) and glycolytic EDL (B) muscles with GH (250 ng/mL). p‐AKT (Ser^473^) and p‐STAT5 (Tyr^694^) protein content following incubation with GH (250 ng/mL) in (C and E) oxidative soleus and (D and F) glycolytic EDL muscles. There were no statisically significant differences among groups. Trending *P* values are indicated with a line between groups. AG, acylated ghrelin; UnAG, unacylated ghrelin; GH, growth hormone; EDL, extensor digitorum longus. Data were analyzed using a repeated measures one‐way ANOVA (*n* = 8 for glucose uptake; *n* = 10 for western blotting).

Because GH is robustly increased in vivo subsequent to ghrelin treatment, we also determined whether GH might directly influence glucose transport in muscle. Growth hormone did not directly alter glucose transport in soleus or EDL muscles, compared to control, untreated muscle (Fig. 7A and B). In soleus, GH did not significantly alter glucose transport in the presence of insulin (*P* = 0.36 compared to insulin alone), although there was an apparent decrease such that this was no longer different from the basal condition (Table 1). AKT phosphorylation was not significantly affected by GH in either oxidative or glycolytic muscles (Fig. 7C and D).

**Table 1:** Effect of growth hormone (250 ng/mL) on glucose transport in the presence of insulin (10 mU/mL)

	Basal	Insulin	Insulin + GH
Soleus	61.6 ± 4.2^a^	92.6 ± 6.8^b^	77.0 ± 9.9^ab^
EDL	54.9 ± 5.6^a^	88.0 ± 9.0^b^	82.6 ± 11.7^ab^

Data are expressed as mean ± standard error, in nmol/g/5 min. Data were analyzed using a repeated measures one‐way ANOVA (*n* = 6). Data sharing a letter are not statistically different from each other. *P* < 0.05 was considered statistically significant.

### GH, but not AG or UnAG, potently increases the phosphorylation of STAT5 (Tyr^694^)

Since some of the effects of ghrelin on peripheral tissues may be due to secondary rise in GH, and we measured glucose transport in the presence of GH, we wanted to verify that GH signaling was present in both muscle fiber types. We examined the phosphorylation of Tyr^694^ of STAT5, which is a common downstream target of GH action and JAK2/STAT signaling (Argetsinger et al. 1993). Phosphorylation of STAT5 Tyr^694^ was significantly elevated in soleus in response to GH (*P* < 0.05), and trended toward an increase in EDL (*P* = 0.08). Acylated and UnAG had no effect on STAT5 phosphorylation in either muscle (Fig. 7E and F).

## Discussion

Given the rapid rise of ghrelin prior to habitual mealtime, it is plausible that ghrelin is involved in the regulation of insulin‐mediated glucose transport in skeletal muscle. We therefore hypothesized that ghrelin would further stimulate insulin‐mediated glucose transport in skeletal muscle, but would not alter glucose transport in the absence of insulin. Our rationale for this was that in the absence of insulin, such an effect of ghrelin in vivo could cause a decrease in blood glucose in the absence of actually consuming food. To our knowledge, the direct effect of ghrelin isoforms on glucose transport in isolated mature skeletal muscle, in the absence of secondary increases in GH, GLP‐1, FFAs, etc., has not been examined. The results of our study demonstrate that neither AG nor UnAG over a wide range of concentrations significantly stimulate glucose transport in either oxidative or glycolytic skeletal muscle, regardless of the presence or absence of insulin. Given that ghrelin administration potently stimulates GH release, we also determined whether GH might have any direct stimulatory effect on muscle glucose uptake that would help to explain the reported in vivo effects of ghrelin. Direct incubation with GH did not significantly increase glucose transport. Although not statistically significant, there was a trend toward a reduction in glucose transport in the presence of insulin following GH incubation, which could potentially explain previous reports of impaired glucose tolerance following ghrelin administration in humans. In agreement with the lack of observed stimulation on muscle glucose transport, AG, UnAG and GH did not affect AKT Ser^473^ or Thr^172^ AMPK. To our knowledge, we are the first to demonstrate an AG‐mediated increase in Thr^287^ CaMKII phosphorylation in muscle, although this clearly was not associated with an increase in glucose transport. Altogether, our findings suggest that any effects of acute ghrelin injection on glucose homeostasis, and in particular postprandial glucose clearance, are not due to the direct effects of ghrelin on skeletal muscle. However, our findings in no way should be taken to discount the potential metabolic effects of chronic ghrelin administration, as this has been shown to produce significant effects on the regulation of glucose metabolism in muscle (Barazzoni et al. 2007; Cappellari et al. 2016).

Skeletal muscle is an important insulin‐responsive tissue involved in the clearance of plasma glucose (DeFronzo et al. 1981) and management of glucose homeostasis. To date there has been sparse data available to suggest ghrelin as a mediator of insulin signaling in muscle. Gershon et al. reported an increase in GLUT4 translocation and glucose uptake in C2C12 muscle cells treated for 24 h with AG (Gershon and Vale 2014). Other groups have reported no change in the phosphorylation of insulin signaling markers, such as AKT, in acute response to ghrelin administration (Vestergaard et al. 2008a). However, the response to chronic ghrelin injections appears to be quite different. Barazzoni and colleagues were among the first to demonstrate that ghrelin can increase key insulin‐responsive proteins, including AKT, GLUT4, and GSK3 (Barazzoni et al. 2007), subsequent to the chronic (4 days) subcutaneous administration of AG. There were no functional assessments of glucose metabolism to indicate whether glucose uptake was also increased in response to ghrelin (Barazzoni et al. 2007). In addition, 4 days of UnAG ghrelin injection has recently been demonstrated to prevent high fat diet‐induced muscle inflammation and preserve normal muscle glucose uptake (Cappellari et al. 2016). Our own data indicates that over a time course of ~1 h (and therefore relevant to the time course of the rapid rise and fall in ghrelin surrounding entrained mealtimes), high physiological concentrations of AG and UnAG ghrelin do not result in enhanced insulin signaling or glucose transport in isolated oxidative or glycolytic skeletal muscle.

Growth hormone's direct effects on muscle glucose transport and AKT phosphorylation were also examined given that AG's effects on glucose metabolism in vivo could potentially be mediated or confounded through the secondary induction of GH. Treatment with GH in differentiated adipocytes ex vivo, as well as in rat skeletal muscle in vivo, suggest that chronically GH can induce insulin resistance as indicated by reductions in IRS‐1/2 tyrosine and AKT phosphorylation and deoxyglucose transport (Smith et al. 1997; Thirone et al. 1997; Takano et al. 2001), which may in part be mediated by an increase in adipose lipolysis and circulating FFAs (Nielsen et al. 2001). Initial studies with ghrelin in humans attempted to control for ghrelin's secondary GH release, using either infusion of somatostatin to inhibit GH release, or GH‐deficient participants (Gauna et al. 2004; Vestergaard et al. 2008a). However, somatostatin is unable to fully blunt significant elevations in serum GH (Vestergaard et al. 2008a), and GH‐deficient individuals often exhibit impaired insulin sensitivity (Hoffman et al. 1994). Our results show that in isolated muscle, GH acutely stimulates STAT5, but does not increase the phosphorylation of AKT, which is consistent with previous findings, some of which indicate GH‐induced reductions in AKT activity (Takano et al. 2001). In line with this, there was no observed functional increase in glucose transport following muscle incubation with a maximal GH dose. Next, we considered whether GH could influence glucose transport in the presence of insulin, as both hormones would be elevated immediately following a meal. Similarly, GH did not alter glucose transport in the presence of insulin; rather, there was a trend toward a reduction in glucose transport although this was not statistically different from our insulin control. Thus, it would seem that any potential stimulatory effect of ghrelin on glucose clearance in vivo is not due to the direct effect of GH on skeletal muscle glucose transport.

Data from human studies suggest that AG administration causes hyperglycemia and insulin resistance, whereas UnAG may be insulin‐sensitizing (Gauna et al. 2004; Vestergaard et al. 2008a). In rodents, animals lacking ghrelin or the ghrelin receptor are resistant from the induction of impaired glucose tolerance and insulin sensitivity induced by a high fat diet (Qi et al. 2011). In contradiction to these findings, Gagnon et al. (Gagnon et al. 2014) demonstrated that AG injection in mice improves blood glucose clearance during an oral glucose tolerance test, an effect which appears to be dependent on the release of the incretin GLP‐1 (Campbell and Drucker 2013). Thus, the role of ghrelin in vivo in the regulation of blood glucose homeostasis is unclear. However, we find the results from Gagnon et al. (Gagnon et al. 2014) intriguing as it suggests a link between ghrelin release and the subsequent metabolism of ingested glucose. Studies directly assessing ghrelin's influence on glucose transport have produced mixed results. In isolated 3T3‐L1 adipocytes and C2C12 myocytes, supramaximal concentrations of both isoforms of ghrelin have been shown to stimulate glucose transport in the absence of insulin (Patel et al. 2006; Gershon and Vale 2014). However, Patel et al. found that only AG, but not UnAG, was able to potentiate insulin's stimulatory effect on glucose uptake in adipocytes (Patel et al. 2006). Unfortunately, there was no assessment of insulin signaling. Although the known ghrelin receptor GHS‐R1a does not appear to be detectable in muscle, ghrelin may act through the CRF‐R2 receptor to stimulate GLUT4 translocation in C2C12 cells, an effect which is blocked in the presence of the selective CRF‐R2 antagonist, anti‐sauvagine‐30 (Gershon and Vale 2014).

Glucose transport in skeletal muscle is mediated through distinct insulin and contraction mediated pathways, and their maximal effects can be additive (Gao et al. 1994). Signals such as Ca^2+^/CaMKII and AMPK appear to be involved in contraction‐mediated GLUT4 translocation and glucose transport (Hayashi et al. 1998; Wright et al. 2004). Ghrelin's effects on these stimulatory processes do not appear to be consistent. For example, neither AG nor UnAG affects the phosphorylation of AMPK in skeletal muscle (Kola et al. 2005); however, in nonskeletal muscle peripheral tissues such as adipose and liver, AG has been shown to inhibit AMPK (Kola et al. 2005). AG has been shown to increase Ca^2+^ release in cardiomyocytes (Sun et al. 2010) and CaMKII activation in glial cells (Chen et al. 2011). To our knowledge, we are the first to report AG‐induced phosphorylation of CaMKII in skeletal muscle. However, the observed increase in CaMKII activation with AG was not accompanied by an increase in glucose transport. Although initial reports by Holloszy indicated that increasing cytosolic Ca^2+^ alone could increase glucose transport (Youn et al. 1991), CaMKII's role in skeletal muscle glucose transport has been recently disputed (Witczak et al. 2010; Jensen et al. 2014). Thus, we speculate that the ghrelin‐induced increase in CaMKII activation observed in this study was not sufficient to increase glucose transport. We have no explanation for the lack of effect of AG on CaMKII phosphorylation in the oxidative soleus. Interestingly, it was the UnAG that tended to increase CaMKII phosphorylation in this muscle type, although the response was variable and not statistically significant. In this study, we did not observe any significant change in AMPK phosphorylation with either AG or UnAG, although there was a trend toward a reduction in the glycolytic EDL with UnAG (*P* = 0.10).

In conclusion, our results indicate that at least acutely, ghrelin does not appear to have any direct stimulatory effects on muscle glucose transport or relevant signaling mechanisms. Further, the lack of a direct effect of GH on glucose transport and signaling (AKT, CaMKII and AMPK) questions the secondary role of GH in directly mediating any acute effects on muscle glucose transport. Further investigation examining any potential direct, acute ghrelin action in other insulin‐sensitive peripheral tissues such as the liver may be important in revealing any short‐term effects of ghrelin responsible for the maintenance of blood glucose homeostasis following the consumption of carbohydrate.

## Conflict of Interest

None declared.
